# Analyzing the Behavior Towards the Use of Interactive Digital Whiteboards for Educational Purposes: A Proposal Based on the Model of Goal-Directed Behavior and the Theory of Planned Behavior

**DOI:** 10.3390/bs14110975

**Published:** 2024-10-22

**Authors:** M. Dolores Gallego, Richard Bagozzi, Salvador Bueno, F. José Racero

**Affiliations:** 1 Department of Management and Marketing, Faculty of Business, Universidad Pablo de Olavide, 41013 Seville, Spain; mdgalper@upo.es (M.D.G.); fjracmon@upo.es (F.J.R.); 2 Ross School of Business, University of Michigan, Ann Arbor, MI 48109, USA; bagozzi@umich.edu

**Keywords:** interactive digital whiteboards, teachers, model of goal-directed behavior, theory of planned behavior, motivations

## Abstract

Information and communication technologies have revolutionized the educational landscape, transforming teaching and learning processes across the globe, and this is the case for interactive digital whiteboards. In particular, this paper focuses on providing a research model to analyze the behavior towards the use of interactive digital whiteboards (IDWs) by teachers in the educational context, highlighting their impact on the intention to use IDWS. The proposed structural equation model is based on the model of goal-directed behavior and the theory of planned behavior, and it is formed by six constructs: (1) attitude, (2) subjective norms, (3) desire, (4) perceived behavioral control, (5) intentions, and (6) behavior. The methodology was adapted to two possible scenarios: (1) positive and (2) negative. The findings show that both theoretical frameworks offer a valid context to explain the motivations that drive the use of IDWs, although there are no significant differences between the two scenarios. Thus, the present article contributes to the existing body of knowledge and provides insights for educators, policymakers, and researchers to leverage the acceptance of IDWs in education. However, some limitations were identified, such as the absence of the point of view of students regarding the use of IDWs, among others.

## 1. Introduction

There is growing interest in knowing whether information technologies (IT) influence student learning [1]. In this context, nowadays, young people use IT in all facets of their lives, and education should not be excluded from this trend [2]. In addition, governments from many countries are providing schools with the most modern IT [3] and many studies have shown that IT investment for educational purposes is a pillar for the development of regions [4,5].

Therefore, educational regulations in many countries indicate that the use of IT in the classroom is an appropriate and valuable didactic means to carry out teaching and learning tasks [6]. Against this background, the use of interactive digital whiteboards (IDWs) has been recently incorporated into the classroom [7], and they are considered as a key IT that directly affects teaching [8]. Therefore, an IDW is a device through which teachers can quickly show educational contents [9]. Indeed, IDWs are considered as significant IT that enhances student motivation [10], so they can be considered as a substitute for traditional blackboards [11]. They have become very popular in educational environments [12,13].

Particularly, IDWs could exert a significant impact on students’ perceptions of engagement [14]. In this context, three advantages can be discerned for students [15]: (1) increasing creativity and use of educational content, (2) improving teaching motivation, and (3) enabling students to be more involved in teaching because they can interact directly with teaching contents. In addition, teachers obtain a real-life view to improve understanding of the complexities and needs of a classroom [16,17].

However, teachers frequently face obstacles that come from (1) other teachers, (2) school administration, (3) technical support, and (4) student factors [18], so they must make an extra effort [19]. In this scenario, the following research question can be posed: what are the variables that explain the application of IDWs in an educational environment? This question guides a twofold objective: (1) to analyze the impact of IDWs in education and (2) to propose a model based on the model of goal-directed behavior and the theory of planned behavior that explains and predicts this impact. This research model, employing the structural equation modeling (SEM) methodology, allows a better understanding of what factors influence teacher behavior towards the use of IDWs.

The paper is organized as follows. Section 2 provides the literature review related to IDWs and presents the research model and the hypotheses; Section 3 develops the research methodology; Section 4 shows the results and the model test; Section 5 discusses the findings and the implications that emerged from the analysis. Finally, Section 6 addresses the conclusions, limitations, and possible directions for future research.

## 2. Literature Review

### 2.1. Theoretical Background

#### 2.1.1. Interactive Digital Whiteboards in the Educational Context

Nowadays, students are considered IT savvy, and consequently, they have sufficient skills to work in interactive and multitasking environments [20]. In this context, IDWs have emerged as an essential educational and collaborative tool in classrooms [7,21,22,23]. Some key features include touch-sensitive screens [24], multimedia integration [25], internet connectivity [26], and integration with other devices, such as computers and smartphones [27].

This set of features allows users to interact with digital content [28]. Indeed, IDWs promote collaborative learning environments by enabling real-time sharing, annotation, and manipulation of content, providing relevant benefits in education, such as (1) enhanced teaching and learning [29], (2) increased student engagement [30], (3) improved collaboration and interaction [7], and (4) access to rich educational resources [31], among others.

In this sense, an IDW is considered a multiskilled platform allowing educators to develop lessons [32] and students to increase their participation and collaboration in class [33], improving student achievement [7,34]. IDWs enhance learning outcomes and skill development [35]. In fact, there are several multimedia and animation elements that can be integrated with IDWs [36], such as videos, images, interactive software, interactive webs, and the metaverse, among others, expanding the scope of learning beyond traditional textbooks [37]. Additionally, IDWs can enhance inclusivity by accommodating diverse learning needs [38], allowing zooming, magnification, text-to-speech, learning disabilities, or language barriers, among others [39].

Moreover, students often exhibit increased motivation, participation, and knowledge retention linked to IDWs and they “can be useful to promote a sense of social engagement among students” [14]. In particular, IDWs support the development of essential 21st-century skills [40], such as critical thinking, collaboration, communication, and digital literacy [41]. Consequently, studies suggest that the use of IDWs in schools imply that teachers need to be trained to use them [42].

Obviously, use of IT is not without its challenges and limitations. One of the most relevant barriers is the high cost [43,44], including the acquisition of hardware, software, necessary infrastructure, technical expertise [45], and high technical complexity [46]. Indeed, teachers may require professional development opportunities to effectively integrate these tools into their lessons [47].

Finally, pedagogical integration is another key issue in the use of IDWs [33]. In this context, educators must design activities and lessons that leverage the interactive capabilities of the whiteboard while aligning with educational objectives [7].

#### 2.1.2. Extended Model of Goal-Directed Behavior

The present study tested an extended model to explain the behavior towards the use of IWDs during teaching in the classroom. This model was based on the model of goal-directed behavior (MGB) [48] and the theory of planned behavior (TPB) [49].

On the one hand, the MGB is a well-recognized framework for examining behavior that suggests people respond rationally in accordance with their (1) attitude toward behavior, (2) subjective normative expectation, and (3) perceived behavioral control. This theory has been used recently in a wide range of topics, from identifying the process of decision formation for environmentally responsible museums [50], to exploring the shopping intentions [51], to predicting crowdfunded behavior for sustainability [52], to determining the motivations of disclosing tourism goals on social media [53], and identifying consumers’ intentions to deploy drones for last-mile delivery [54], among others. In particular, the application of MGB in the educational field is scarce and it is beginning to be used in this context; for example, to determine the potential behavioral consequences of expectations on school performance [55], to explain users’ continuance intention towards e-learning [56], to explain their intention to use MOOCs [57], to explain the intention to use mobile English learning services [58], to analyze teachers’ intention to use technology-enabled learning [59], and to study how experiences of learning with IT can affect teachers’ intentions to use it [60].

Nevertheless, no works have used this framework to analyze the impact of IDWs in education and how their use supports the educational goals of teachers. Considering that teachers attempt to develop themselves in improving teaching methods [61], we developed a model that explains and predicts the impact of the use of IDWs as a tool for teachers.

This framework, proposed by [48], posits that human actions are driven by specific goals or objectives [62]. This theory differs from the previous models (theory of planned behavior (TPB) and the theory of reasoned action (TRA)) in some aspects [48]: (1) specifically, it includes the desire to carry out a behavior (or the desire for a goal) as a reflection of the incorporation and transformation of previous effects of attitude, subjective norms, perceived behavioral control, and anticipated emotions (AEs), influencing the intention to enact this behavior; (2) it formulates anticipated emotions based on prefectural judgments that each person makes of the anticipated achievement of a goal or its anticipated failure, which changes from time to time, depending on the context; and (3) is based on the relevance of using unipolar items in the measurement of AEs, compared to the bipolar items of other theoretical approaches, as more used by psychologists [63].

On the other hand, since the MGB is inspired by TPB, where the latter is a subset of the former, it is inevitable that the approaches share theoretical content. Indeed, the TPB continues to be a foundational framework in understanding and predicting human behavior [64] and has contributed significantly to a wide range of fields, such as health [65,66], education [67,68,69], tourism [70,71] and IT [72,73], among others. As the theory evolves and adapts to new research findings and critiques, it remains a valuable tool for shaping interventions and strategies aimed at fostering positive behavioral change [74], among other aspects.

### 2.2. Research Model and Hypotheses

**Figure 1:**
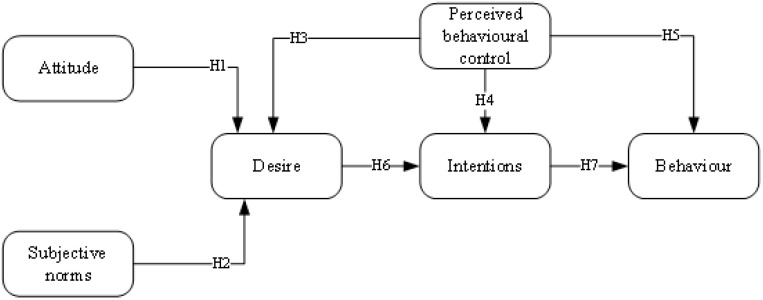
Proposed research model.

The proposed research model offer a comprehensive lens through which to understand the intricate relationship between goals, beliefs, desires, intentions, and behavior [75,76]. By recognizing the underlying processes that drive human behavior, researchers and practitioners can use both frameworks (MGB and TPB) to facilitate positive changes and empower individuals to achieve their aspirations [77]. Specifically, this proposal aims to explain and predict the impact of the use of IDWs as a tool for teachers. Based on this purpose, Figure 1 shows the proposed research model and hypotheses.

The scope of evidence to answer these hypotheses is relevant because it allows us to understand the factors that influence behavior towards the use of IDWs. In fact, the present study will allow us to fill the gaps in the literature regarding the factors that motivate the use of this IT. Specifically, examining how attitude, subjective norms, and perceived behavioral control affect desire and intention to use helps to identify key motivators in the adoption of IDWs. In addition, this research proposal provides a comprehensive view of the steps that users take from intention to action, which can guide effective strategies for the adoption and development of IDWs.

#### 2.2.1. Relationship Between Attitude and Subjective Norm and Desire to Use IDWs

Unlike TPB, desire is considered a crucial construct by MGB that allows one to explain the formation of personal decisions [78]. In fact, this dimension is not present in TPB. Concretely, according to [79], in TPB the constructs of attitude, subjective norms, and perceived behavioral control impact in a direct way on intentions; however, in MGB these same dimensions affect intentions through desire [48]. Desire integrated the effects of the antecedents, transforming their influence on intentions. After incorporating desire into MGB, it is possible to observe a significant increase in the explanatory capacity of the model to predict behaviors in comparison to TPB [52]. In addition, MGB assumes that past behavior or habits are significant predictors of desire, intention, and human behaviors [48,80]. In this manner, desire could be referred in our study to the psychological motivations that incentivized the intention to use IDWs for educational purposes.

In this context, the activation of the desire could be possible when teachers feel able to use IDWs for teaching. Thus, teachers’ motivational inputs could come from the motivation to adapt to this IT. Indeed, these motivational inputs can directly impact intention without being completely driven by desires.

Under these premises, attitude could play a relevant role in the activation of the desire and refers to the degree to which an individuals has feelings (favorable or unfavorable) about the behavior [49]. In fact, Ref. [81] defined attitude as “a disposition to respond favorably or unfavorably towards some psychological object”. In that context, attitude proved to have a significant impact and positive effect on intentions [50] in general domains [49,82] and technology-based learning [83,84]. Accordingly, the present study incorporates teachers’ positive or negative perceptions towards using IDW in their classroom. Thus, a positive relationship between attitude and desire was hypothesized (H1). The formulation of this hypothesis would be as follows:

**H1:**  *Attitude has a positive effect on desire to use IDWs*.

By contrast, subjective norms refers to the perceived social pressure to perform or not to perform the behavior [49]. In fact, the concept of subjective norms is also known as a kind of social influence since it is related to the belief that one has about the reaction of others to one’s own behavior [85] and, in this manner, it could play an important role in initial acceptance. In this respect, according to [86] (p. 279) “subjective norms capture the interpersonal aspect of behavior and reflect the impact of directly felt expectations from other people which are largely based on the need for approval”. In addition, some researchers [48,86] have determined that subjective norms do not directly fortify an individual’s intentions, but affect intentions indirectly through desire. Consequently, subjective norms exert a significant influence on desire. Based on these assumptions, this study posits that subjective norms have a positive influence on desire (H2).

**H2:**  *Subjective norms have a positive effect on desire to use IDWs*.

#### 2.2.2. Perceived Behavioral Control

MGB identifies perceived behavioral control as a relevant dimension [87] in order to highlight that control beliefs can also influence individual behavior [88] and it shows the perception that people have about the ease or difficulty of carrying out the behavior of interest [49]. This construct is referred to as “an individual’s confidence or ability to perform a specific behavior and it is considered to be an imperative factor of intentions and actual behavior” [78] (p. 1420). Specifically, it tries to explain how people behave in relation to a personal desire in situations of difficulty or control [89]. In this manner, a significant link between perceived behavioral control and desire was found in a previous study [48], which allows us to propose the following hypothesis.

**H3:**  *Perceived behavioral control has a positive effect on desire to use IDWs*.

Moreover, MGB posits that perceived behavioral control positively impacts the decision to perform a specific behavior in a decision-making context [90]. In this manner, grounded on the MGB, perceived behavioral control contributes to incentives to adopt behavioral intentions and a certain behavior [48]. Consequently, the two following hypotheses are defined.

**H4:**  *Perceived behavioral control has a positive effect on intentions to use IDWs*.

**H5:**  *Perceived behavioral control has a positive effect on behavior towards the use of IDWs*.

#### 2.2.3. Desire, Intentions, and Behavior

Based on the proposed research model, desire acted as a mediator between subjective norms and attitude and behavioral intention. Desire was incorporated from MGB in order to capture the motivations that exists between antecedents and intentions [76]. Thus, it is the most proximal determinant of behavioral intentions [91]. In this manner, while the concept of desire is related to verbs such as to wish or to want, intentions are linked with the verbs to plan or to decide [92]. This positive relationship has been widely demonstrated in studies from many fields [51,93,94,95,96,97].

Transferring this relationship to our study, teachers with a strong desire to use IDWs during their class should experience a motivational impetus to perform an intention oriented to use IDWs. Therefore, a teacher’s intentions, as well as their knowledge and skills about IDWs, should predict actual behavior [81]. In this context, behavior would be referred to the teachers’ IDW adoption. Our proposal assumes that intentions significantly enhance the predictive behavior [98]. In this respect, Ref. [99] asserted that an individual develops emotions based on an expected outcome from a behavior before performing the actual behavior. Thus, all these assumptions establish the foundations to define the two last hypotheses:

**H6:**  *Desire has a positive effect on intentions to use IDWs*.

**H7:**  *Intentions have a positive effect on behavior towards the use of IDWs*.

## 3. Research Methodology

A structural equation model (SEM) was proposed to test the hypotheses. In particular, the analysis was performed in two stages. First, the descriptive statistics and the reliability of the measurement were analyzed with the statistical software SPSS 27. Second, the SEM was testing through the software package LISREL v.12 in order to complete correlations a factor analysis, and the path analysis equation model.

### 3.1. Questionnaire and Sample

All constructs in this study were measured with multiple items after an extensive literature review. Most of the items that measure the study variables were adapted from [48], although more recent studies have been taken into account as well [100,101].

This study was carried out at secondary schools (teachers) from the Public Education System of Andalusia (Spain). This region was selected because the government of Andalusia exercises regulatory and executive power in education, and recently it has boosted some political initiatives to incentive the use of IWDS in all grades.

Specifically, a questionnaire was proposed with 25 items (Appendix A) organized into two sections: (1) demographic variables and (2) research model variables. In addition, the study adapted to two possible scenarios: (1) positive and (2) negative, with two different introductions (positive and negative) that explain the development of both scenarios. The positive scenario proposed a story that expresses a very positive experience with IDWs, and it was focused on the benefits of its use. By contract, the negative scenario provided a boring or bland description about the use of IDWs. The intention of this design was to seek the formation of two large groups of respondents to the same questionnaire, a web-based survey developed with the online survey tool LimeSurvey, but with different scenarios.

The respondents indicated their agreement or disagreement with the above items using a five-point Likert-type scale, ranging from “strongly disagree” (1) to “strongly agree” (5), or (1) “does not describe me at all” to (5) “describes me very well”, with (3) “describes me moderately well”, according to the type of question. The survey was conducted from May to December 2022. A total of 1020 questionnaires were received, with 609 valid and complete. Specifically, 293 came from the negative scenario and 316 from the positive.

### 3.2. Profile of the Respondents

In the first part of the questionnaire, the responses to four demographic dimensions were required. These questions yielded the profile of the respondents (Table 1).

In this manner, the educators consulted were mostly female (181 male and 428 female). This aspect is especially relevant because it is frequent that the samples of studies related to IT are biased more towards the male gender; besides, it is highlighted that most of the respondents were over 30 years of age and have had extensive experience as teachers when we look at the years they have dedicated to this profession.

## 4. Results

### 4.1. Descriptive Statistics

This analysis started with the evaluation of the internal consistency of the results (Table 2) for both scenarios. This measure based on the correlations between items tries to identify if there are some items that measure the same construct. In addition, an exploratory factor analysis (EFA) of the different scales was carried out for all the constructs using a Kaiser’s Varimax Rotation. This analysis demonstrates the unidimensionality of the scales (measure a single trait) of all the constructs in the study [102] in both scenarios. In this way, the selected extraction method was principal axis factoring [78].

Cronbach’s α reliability coefficients were between 0.85 and 0.96, which is well above the minimum acceptable threshold of 0.70 [103]. Therefore, the measurement instruments were deemed reliable gauges of the constructs. This value was obtained after taking out of the model items SN3, SN4, and DS3 because they were below the previously indicated threshold of 0.7.

Additionally, the discriminant validity for the measurement model was assessed in order to determine that each factor represented a separate dimension in the positive and negative scenarios (Table 3). In this analysis, the square roots of the AVEs were greater than 0.50 and greater than the absolute values of the off-diagonal elements in the corresponding rows and columns of the correlation matrix. In this manner, these results suggest that all the constructs have convergent validity [104].

### 4.2. Model Fit

LISREL v. 12 was used to assess the model fit. Table 4 gathers the main fit indices for the proposed research model along with the recommended thresholds. In particular, according to [105], these results demonstrate that the research model has a satisfactory fit over the formulated thresholds for both the positive and negative models.

### 4.3. Test of Hypotheses

**Figure 2:**
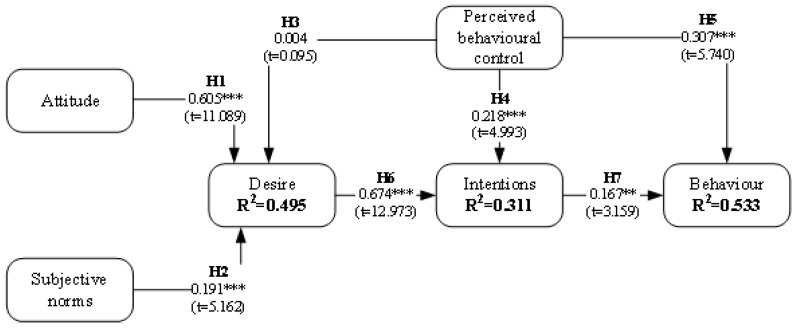
Structural model results (positive scenario). ** *p* < 0.01; t (0.01) = 2.5904; *** *p* < 0.001; t (0.001) = 3.3195.

**Figure 3:**
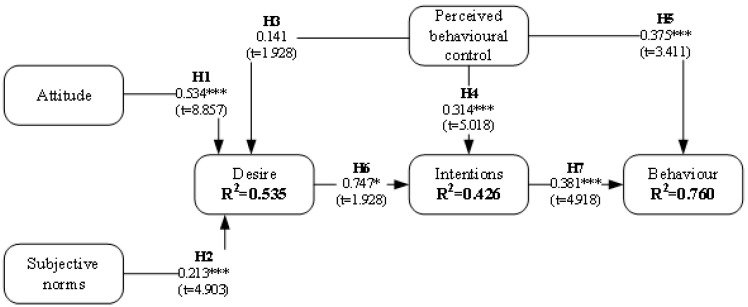
Structural model results (negative scenario). * *p* < 0.05; t (0.05) = 1.9670; *** *p* < 0.001; t (0.001) = 3.3195.

The research model was tested using LISREL 12.0 with an estimation method of maximum likelihood [106]. This test was completed for both the positive (Figure 2) and negative (Figure 3) models.

Table 5 contains a synthesis of the test, demonstrating the statistical significance of most hypotheses. In this manner, in both models, all hypotheses were supported for *p* < 0.05, *p* < 0.01, *p* < 0.001, except Hypothesis 3, which defined a relationship between PBC and DES. Thus, this result determines that PBC is not a predictor of DES. In this way, H1, H2, H4, and H5 for both scenarios were supported with a high significance (*p* < 0.001). In addition, while H6 was widely supported for the positive scenario (β = 0.674, *p* < 0.001), the test supported it with a lower, but acceptable, significance (β = 0.747, *p* = 0.05) for the negative scenario. Finally, H7 had a strong confirmation in the negative scenario (β = 0.167, *p* < 0.001), although for the positive it was slightly less, but acceptable (β = 0.167, *p* < 0.01).

## 5. Discussions

This article presents a study to analyze the connections between the behavior towards the use of IDWs in education and the constructs (1) attitude, (2) subjective norms, (3) desire, (4) perceived behavioral control, and (5) intentions. These dimensions were extracted from two theoretical frameworks. In particular, the results achieved have shown that both theories offer a valid context to explain the motivations that drive the use of IDWs for educational purposes as a multiskilled IT to develop flexible lessons, mainly incorporating multimedia resources, educational websites, and digital libraries, among others. In addition, the research was designed considering two possible scenarios (positive and negative), and the same hypotheses have been tested in both.

In this way, the findings identify some constructs which explain the behavior towards the use of IWDs. This proposal was based on anterior studies [48,75,80,96,98,107]. In particular, the behavior is broadly explained by the research model showing that it is predicted by the rest of the constructs. Specifically, the model explained 53.3% of the variance in behavior for the positive scenario and 76% for the negative. Moreover, the variance in desire was explained by the model with 49.5% variance for the positive scenario and 53.5% for the negative. Likewise, the model for the positive scenario describes 31.1% of intentions and 42.6% for the negative. These results were much superior to those obtained by other studies that designed models with the same theoretical frameworks [50,93,96].

However, it can also be deduced from these results that the present research does not explain a large percentage of behavior towards IDWs because the model has focused on factors such as attitude and subjective norms following the TPB and MGB theoretical frameworks. This implies a simplification of the analysis within the context of IDW use. Consequently, it is acknowledged that other relevant factors have been left out of the study, the inclusion of which would have considerably broadened the scope of the study. Therefore, one of the key limitations of the study is that it does not cover all possible dimensions that could influence the use of IDWs. A possible suggestion for future research would be to include models such as TPACK or UTAUT to provide a more holistic and integrated view of the factors that affect the adoption and use of these tools. This would allow for a more robust analysis that includes the interaction of pedagogical and technological knowledge, providing a more complete understanding of the determinants of their use.

In keeping with previous research, the proposed research model offers a comprehensive framework to explain the factors that determine the behavior towards the use of IDWs by teachers. In fact, these findings are broadly in line with previous studies. In this way, according to [48], the attitudes previously experienced by individuals have an important effect on the desire construct. In a similar way, Ref. [75] identified attitude towards behavior as the degree to which an individual has evaluations (favorable or unfavorable) about the behavior; attitude proved to have a significant impact and positive effect towards intentions [50]. The results significantly predicted in Hypotheses 1 and 2 have been tested with the analyses carried out. Thus, the present study reveals that the desire to use IDWs in the classroom is explained by attitude and subjective norms, the latter being understood as the perceived social pressure to perform or not to perform the behavior [75,86].

In addition, the construct of perceived behavioral control had a significant impact on two of the central constructs of the model (intentions and behavior), although not on desire. These results are similar to finding in different contexts in the literature. Indeed, according to [87], perceived behavioral control is considered a remarkable variable in the decision-making process due to its impact on individual behavior. Regarding this construct, Ref. [75] posited that perceived behavioral control reflects the individual estimation about the ease or difficulty of developing a specific behavior. Consequently, it should stimulate the desire to use IDWs, but in this case no significant influence on the desire has been observed. Therefore, Hypothesis 3 cannot be affirmed. Although MGB and TPB provide a priori useful conceptual frameworks for interpreting this connection, this result could be explained in several ways. Frist, from a practical point of view, in this research context IWDs are being introduced in the educational centers of the Andalusian Public System, where the participants of this study come from. In this respect, the setting-up of IDWs corresponds to political decisions in which factors that could affect the acceptance of this IT by its potential users, in this case teachers, are not considered. This way of incorporation of IWDs could be perceived by teachers as imperative and, therefore, could not stimulate the desire to use them. Second, from a theoretical perspective, the lack of relationship between the PBC and desire can be explained by the emotional nature of desire itself. While the PBC assesses the ability to carry out a behavior, desire is more associated with emotional motivation and affective factors, which do not always depend on the perception of control. Instead, the relationships with intentions and behavior were supported, as expected [48], affirming that perceived behavioral control activates intentions and behavior.

Additionally, on the one hand, the findings have shown that desire acted as a mediator between subjective norms and attitude and intentions (Hypothesis 6), indicating that highly motivated teachers have a greater desire to use IDWs for educational purposes. On the other hand, teachers’ intentions predict the actual behavior (Hypothesis 7) as it was proposed by [81,99].

These discussions bring some implications. From a theoretical angle, this study contributes to improve the knowledge about the adoption of IT in schools, in particular, IDWs. Indeed, these research findings identify the psychological conditions among educators needed to develop future proposals through which to assess the intention to use IDWs in schools. Therefore, adopting a managerial or policy view, the present study offers to governments and management committees of educational centers a clear group of dimensions on which to design policies that boost the acceptance of IT among teachers. In addition, this type of study supports the convenience of implementing IT in educational centers as tools to assist the development of lessons.

## 6. Conclusions

This work has focused on developing a research model that allows testing of behavior towards use of IDWs in education. Specifically, the proposal has combined a set of constructs from MGB and TPB in order to reach its main objectives: (1) to analyze the impact of IDWs in education and (2) to propose a model that explains and predicts this impact. In this context, this research article attempted to analyze some of the factors that influence the adoption of IWDs. Thus, it has been revealed that studying the motivations that drive the use of recent IT in education is always intriguing, although the literature on this topic is not very extensive in the field of IWDs. The present study has been an effort to amend this.

The analyses carried out clearly show that the proposed model has a high explanatory capacity for the behavior of teachers towards the use of IWDs compared to other studies with similar objectives and fields. It is notable that the variables that explain the motivations that encourage the use of IWDs in class by teachers have been identified. These are referred to as (1) attitude, (2) subjective norms, (3) desire, (4) perceived behavioral control, and (5) intentions. Thus, these results could be especially useful for teachers and educational institutions in order to discover the technological acceptance mechanisms regarding IDWs.

Specifically, conducting studies such as this one provides three major benefits. First, the results help to understand that motivational factors are fundamental in designing training strategies and programs that promote the effective adoption of technologies in the classroom by improving the teaching and learning experience. Second, these findings can guide the creation of more successful public policies, ensuring that resources invested in educational technology are used effectively and that interventions are aligned with teachers’ needs and motivations. Finally, it has been shown that motivational factors are capable of increasing teachers’ willingness and confidence to use interactive technologies in their classes, which in turn can improve educational outcomes.

However, this study is not free of limitations. First, future works should expand the number of variables to increase the explanatory capacity of the model. Although the model explains a high percentage of the variance in behavior, it does not completely predict it. Second, the results have not revealed major differences between the positive and negative scenarios, so it has not been possible to delve into the factors that encourage or discourage the use of IWDs. Finally, this study has not considered the student’s perspective. From this point of view, it would be interesting to undertake new studies that allow us to compare the motivational differences between both groups when they are using IWDs.

## Figures and Tables

**Table 1:** Demographic profiles of the respondents.

Characteristic		Positive	Negative	(%)
Gender	Male	94	87	29.72%
	Female	222	206	70.28%
Age	Under 25	2	3	4.93%
	Between 25 and 30	27	28	15.27%
	Between 30 and 40	60	66	30.87%
	Between 40 and 50	137	128	32.84%
	Between 50 and 60	81	63	14.12%
	Over 60	9	5	1.48%
Years of experience	Under 5	66	67	18.39%
	Between 5 and 10	54	46	17.57%
	Between 10 and 15	52	53	16.42%
	Between 15 and 20	47	48	14.45%
	Between 20 and 25	50	41	14.45%
	More than 25	47	38	7.72%
Taught most	Kindergarten	120	19	31.36%
	Elementary School		71	16.91%
	Middle School	131	103	27.91%
	High School	29	39	5.75%
	Vocational Training Basic Degree	3	6	1.97%
	Intermediate Vocational Training	18	9	7.55%
	Higher Level Vocational Training	10	28	2.13%
	University	1	3	2.63%
	Other Teaching	4	15	0.66%

**Table 2:** Summary of measurement scales.

	Positive							Negative						
Construct	Mean	Std. Dev.	Lambda Stand.	Factor Loading	Composite Reliability	AVE	Cronbach’s α	Mean	Std. Dev.	Lambda Stand.	Factor Loading	Composite Reliability	AVE	Cronbach’s α
ATT					0.930	0.815	0.929					0.940	0.839	0.938
ATT 1	4.21	0.977	0.930	0.921				4.22	0.959	0.919	0.912			
ATT 2	4.08	0.961	0.847	0.848				4.04	1.038	0.887	0.885			
ATT 3	4.22	0.982	0.929	0.938				4.22	0.953	0.941	0.948			
SN					0.946	0.898	0.945					0.916	0.845	0.914
SN1	2.93	1.283	0.981	0.946				3.08	1.394	0.878	0.919			
SN2	2.99	1.275	0.913	0.946				3.32	1.412	0.959	0.916			
PBC					0.858	0.669	0.850					0.737	0.490	0.754
PBC1	3.31	1.443	0.791	0.771				3.65	1.439	0.567	0.680			
PBC2	3.72	1.416	0.896	0.942				4.13	1.216	0.662	0.822			
PBC3	3.70	1.237	0.760	0.724				4.01	1.138	0.843	0.655			
DES					0.947	0.900	0.947					0.972	0.945	0.969
DES1	4.25	0.997	0.959	0.949				4.33	1.005	0.963	0.969			
DES2	4.16	1.072	0.938	0.949				4.28	1.048	0.977	0.969			
INT					0.886	0.722	0.883					0.899	0.749	0.897
INT1	3.64	1.131	0.821	0.807				3.67	1.325	0.824	0.823			
INT2	3.70	1.196	0.905	0.933				3.84	1.282	0.905	0.915			
INT3	3.72	1.086	0.821	0.802				3.94	1.189	0.865	0.854			
BI					0.960	0.890	0.960					0.964	0.898	0.963
BI1	4.00	1.125	0.925	0.922				4.16	1.059	0.967	0.967			
BI2	3.99	1.138	0.969	0.973				4.13	1.098	0.966	0.967			
BI3	4.07	1.097	0.935	0.933				4.24	1.032	0.909	0.906			

**Note.** ATT: attitude; SN: subjective norms; PBC: perceived behavioral control; DES: desire; INT: intentions; B: behavior.

**Table 3:** Discriminant validity of the constructs.

	Positive						Negative					
	ATT	SN	PBC	DES	INT	BI	ATT	SN	PBC	DES	INT	BI
ATT	**0.664**						**0.704**					
SN	0.349	**0.806**					0.440	**0.714**				
PBC	0.317	0.196	**0.446**				0.464	0.480	**0.240**			
DES	0.662	0.453	0.241	**0.810**			0.659	0.541	0.471	**0.893**		
INT	0.224	0.109	0.451	0.156	**0.522**		0.368	0.343	0.489	0.477	**0.561**	
BI	0.512	0.325	0.382	0.673	0.350	**0.791**	0.584	0.508	0.588	0.825	0.513	**0.806**

**Note.** ATT: attitude; SN: subjective norms; PBC: perceived behavioral control; DES: desire; INT: intentions; BI: behavior. Diagonal elements (in bold) are the square root of the shared variance between the constructs and their measures (square root of AVE).

**Table 4:** Overall fits of models.

Fit Index	Positive	Negative	Recommended Value
χ2/Grade of Freedom	1.004	1.057	≤3.00
Non-Normed Fit Index (NNFI)	0.948	0.951	≥0.90
Comparative Fit Index (CFI)	0.960	0.962	≥0.90
Root Mean Square Error of Approximation (RMSEA)	0.0731	0.0743	≤0.05

**Table 5:** Results of the structural equation modeling.

Hypothesis (Path)	Positive			Negative		
	Path Coefficient	t-Value	Supported	Path Coefficient	t-Value	Supported
H1: ATT→DES	0.605	11.089 ***	Yes	0.534	8.857 ***	Yes
H2: SN→DES	0.191	5.162 ***	Yes	0.213	4.903 ***	Yes
H3: PBC→DES	0.004	0.095	No	0.141	1.928	No
H4: PBC→INT	0.218	4.993 ***	Yes	0.314	5.018 ***	Yes
H5: PBC→B	0.307	5.740 ***	Yes	0.375	3.411 ***	Yes
H6: DES→INT	0.674	12.973 ***	Yes	0.747	1.928 *	Yes
H7: INT→B	0.167	3.159 **	Yes	0.381	4.918 ***	Yes

**Note.** ATT: attitude; SN: subjective norms; PBC: perceived behavioral control; DES: desire; INT: intentions; B: behavior. Significant at: * *p* < 0.05; t (0.05) = 1.9670; ** *p* < 0.01; t (0.01) = 2.5904; *** *p* < 0.001; t (0.001) = 3.3195.

## Data Availability

The data presented in this study are available on request from the corresponding author.

## Appendix A. Questionnaire

**Constructs**	**Items**	**Sources**
Attitude (ATT)	My attitude or evaluation of using the IDW can be best summarized as: ATT1 …a good idea. ATT2 …a wise idea. ATT3 …a favorable idea.	Ajzen and Fishbein (1977) [98] Perugini and Bagozzi (2001) [48]
Subjective Norms (SN)	Most people who are important to me (teachers and students): SN1 …think I should use IDWs during my class. SN2 …would want me to use IDWs during my class. SN3 …support my participation in using IDWs during my class. SN4 …approve my participation in using IWDs during my class.	Ajzen and Fishbein (1977) [98] Perugini and Bagozzi (2001) [48]
Perceived behavioral control (PBC)	PBC1. Whether I use IDWs or not is completely up to me. PBC2. I am confident that if I want, I can use IDWs during my class. PBC3. It is easy for me to use the IDWs during my class.	Perugini and Bagozzi (2001) [48] Lee et al. (2012) [107] Kim and Preis (2016) [96]
Desire (DES)	DES1. I want to use IDWs in my class. DES2. I desire to use IDWs in my class. DES3. I feel an urge to use IDWs in my class.	Perugini and Bagozzi (2001) [48] Lee et al. (2012) [107] Kim and Preis (2016) [96]
Intentions (INT)	INT1. I carefully check information about IDWs before use in class. INT2. I read and check all digital content before using it in the IDW. INT3. I diligently prepare my class according to the use of IDWs.	Perugini and Bagozzi (2001) [48] Lee et al. (2012) [107] Kim and Preis (2016) [96]
Behavior (B)	BI1. I intend to use IDWs on a regular basis in my classes. BI2. I plan to use IDWs in my classes frequently. BI3. I expect that I will use regularly IDWs in my classes.	Perugini and Bagozzi (2001) [48] Lee et al. (2012) [107] Kim and Preis (2016) [96]

## References

[1] Borgonovi F., Pokropek M., Pokropek A. (2023). Relations between Academic Boredom, Academic Achievement, ICT Use, and Teacher Enthusiasm among Adolescents. Comput. Educ..

[2] Liong M., Yeung D.Y., Cheng G.H.-L., Cheung R.Y.H. (2023). Profiles of ICT Identity and Their Associations with Female High School Students’ Intention to Study and Work in ICT: A Mixed-Methods Approach. Comput. Educ..

[3] Fernández-Gutiérrez M., Gimenez G., Calero J. (2020). Is the Use of ICT in Education Leading to Higher Student Outcomes? Analysis from the Spanish Autonomous Communities. Comput. Educ..

[4] Shobande O.A., Asongu S.A. (2022). The Critical Role of Education and ICT in Promoting Environmental Sustainability in Eastern and Southern Africa: A Panel VAR Approach. Technol. Forecast. Soc. Chang..

[5] Zafar M.W., Zaidi S.A.H., Mansoor S., Sinha A., Qin Q. (2022). ICT and Education as Determinants of Environmental Quality: The Role of Financial Development in Selected Asian Countries. Technol. Forecast. Soc. Chang..

[6] Vargas-Montoya L., Gimenez G., Fernández-Gutiérrez M. (2023). ICT Use for Learning and Students’ Outcomes: Does the Country’s Development Level Matter?. Socioecon. Plan. Sci..

[7] Reguera E.A.M., Lopez M. (2021). Using a Digital Whiteboard for Student Engagement in Distance Education. Comput. Electr. Eng..

[8] Tortorella G.L., Narayanamurthy G., Sunder M.V., Cauchick-Miguel P.A. (2021). Operations Management Teaching Practices and Information Technologies Adoption in Emerging Economies During COVID-19 Outbreak. Technol. Forecast. Soc. Chang..

[9] Remón J., Sebastián V., Romero E., Arauzo J. (2017). Effect of Using Smartphones as Clickers and Tablets as Digital Whiteboards on Students’ Engagement and Learning. Act. Learn. High. Educ..

[10] Hassanuddin N.A., Yusoff S., Noh N.H.M., Sukri N.M. (2022). The Use of Interactive Whiteboard in Teaching Mathematics and Statistics. Int. J. Adv. Res. Educ. Soc..

[11] Higgins S., Beauchamp G., Miller D. (2007). Reviewing the Literature on Interactive Whiteboards. Learn. Media Technol..

[12] Haldane M. (2007). Interactivity and the Digital Whiteboard: Weaving the Fabric of Learning. Learn. Media Technol..

[13] Mercer N., Hennessy S., Warwick P. (2010). Using Interactive Whiteboards to Orchestrate Classroom Dialogue. Technol. Pedagog. Educ..

[14] Campbell M., Detres M., Lucio R. (2019). Can a Digital Whiteboard Foster Student Engagement?. Soc. Work Educ..

[15] Jones K. (2004). Using Interactive Whiteboards in the Teaching and Learning of Mathematics: A Research Bibliography. Micromath.

[16] Al-Qirim N. (2011). Determinants of Interactive White Board Success in Teaching in Higher Education Institutions. Comput. Educ..

[17] Armstrong V., Barnes S., Sutherland R., Curran S., Mills S., Thompson I. (2005). Collaborative Research Methodology for Investigating Teaching and Learning: The Use of Interactive Whiteboard Technology. Educ. Rev..

[18] Alfaki M., Khamis A.H.A. (2014). Difficulties Facing Teachers in Using Interactive Whiteboards in Their Classes. Am. Int. J. Soc. Sci..

[19] Gosain K. (2016). Factors Influencing the Use of Interactive Whiteboard. Int. J. Adv. Res..

[20] Szymkowiak A., Melović B., Dabić M., Jeganathan K., Kundi G.S. (2021). Information Technology and Gen Z: The Role of Teachers, the Internet, and Technology in the Education of Young People. Technol. Soc..

[21] Chen I.-H., Gamble J.H., Lee Z.-H., Fu Q.-L. (2020). Formative Assessment with Interactive Whiteboards: A One-Year Longitudinal Study of Primary Students’ Mathematical Performance. Comput. Educ..

[22] Kunz A., Nescher T., Küchler M. CollaBoard: A Novel Interactive Electronic Whiteboard for Remote Collaboration with People on Content. Proceedings of the 2010 International Conference on Cyberworlds.

[23] Teng M.F. (2021). Interactive-Whiteboard-Technology-Supported Collaborative Writing: Writing Achievement, Metacognitive Activities, and Co-Regulation Patterns. System.

[24] Bilý J., Miština J. (2023). Using an Interactive Whiteboard to Increase the Effectiveness of Teaching at Secondary Schools. RE-SOURCE.

[25] Joshi D.R., Adhikari K.P., Khanal J., Belbase S., Khanal B. (2023). Developing and Integrating Digital Resources in Online Mathematics Instruction and Assessment during COVID-19. Cogent Educ..

[26] Zhou Y., Li X., Wijaya T.T. (2022). Determinants of Behavioral Intention and Use of Interactive Whiteboard by K-12 Teachers in Remote and Rural Areas. Front. Psychol..

[27] Kumar R., Selva Ganesh R. (2022). Dealing with Online and Blended Education in India. DECISION.

[28] Hennessy S., Deaney R., Ruthven K., Winterbottom M. (2007). Pedagogical Strategies for Using the Interactive Whiteboard to Foster Learner Participation in School Science. Learn. Media Technol..

[29] Alvarez C., Salavati S., Nussbaum M., Milrad M. (2013). Collboard: Fostering New Media Literacies in the Classroom through Collaborative Problem Solving Supported by Digital Pens and Interactive Whiteboards. Comput. Educ..

[30] López O.S. (2010). The Digital Learning Classroom: Improving English Language Learners’ Academic Success in Mathematics and Reading Using Interactive Whiteboard Technology. Comput. Educ..

[31] Santarosa L., Conforto D., Machado R.P. (2014). Whiteboard: Synchronism, Accessibility, Protagonism and Collective Authorship for Human Diversity on Web 2.0. Comput. Hum. Behav..

[32] Haleem A., Javaid M., Qadri M.A., Suman R. (2022). Understanding the Role of Digital Technologies in Education: A Review. Sustain. Oper. Comput..

[33] Mhlongo S., Mbatha K., Ramatsetse B., Dlamini R. (2023). Challenges, Opportunities, and Prospects of Adopting and Using Smart Digital Technologies in Learning Environments: An Iterative Review. Heliyon.

[34] Bautista-Vallejo J.M., Hernández-Carrera R.M., Moreno-Rodriguez R., Lopez-Bastias J.L. (2020). Improvement of Memory and Motivation in Language Learning in Primary Education through the Interactive Digital Whiteboard (IDW): The Future in a Post-Pandemic Period. Sustainability.

[35] Haldane M. (2010). A New Interactive Whiteboard Pedagogy through Transformative Personal Development. Interactive Whiteboards for Education: Theory, Research and Practice.

[36] Dwivedi Y.K., Hughes L., Baabdullah A.M., Ribeiro-Navarrete S., Giannakis M., Al-Debei M.M., Dennehy D., Metri B., Buhalis D., Cheung C.M.K. (2022). Metaverse beyond the Hype: Multidisciplinary Perspectives on Emerging Challenges, Opportunities, and Agenda for Research, Practice and Policy. Int. J. Inf. Manag..

[37] Šumak B., Šorgo A. (2016). The Acceptance and Use of Interactive Whiteboards among Teachers: Differences in UTAUT Determinants between Pre- and Post-Adopters. Comput. Hum. Behav..

[38] Freire A.P., Linhalis F., Bianchini S.L., Fortes R.P.M., Pimentel M. (2010). Revealing the Whiteboard to Blind Students: An Inclusive Approach to Provide Mediation in Synchronous e-Learning Activities. Comput. Educ..

[39] Bakadam E., Asiri M.J.S. (2012). Teachers’ Perceptions Regarding the Benefits of Using the Interactive Whiteboard (IWB): The Case of a Saudi Intermediate School. Procedia-Soc. Behav. Sci..

[40] FWA H.L. Investigating Collaborative Problem Solving Temporal Dynamics Using Interactions within a Digital Whiteboard. Proceedings of the 15th International Conference on Computer Supported Education.

[41] Varas D., Santana M., Nussbaum M., Claro S., Imbarack P. (2023). Teachers’ Strategies and Challenges in Teaching 21st Century Skills: Little Common Understanding. Think. Ski. Creat..

[42] AlAdl A.M., Ahmed A.M. (2019). The Effects of Using an Electronic Interactive Whiteboard in Developing Students’’ Attitude, Cognitive Motivation and Academic Achievement. J. Educ. Pract..

[43] Slay H., Siebörger I., Hodgkinson-Williams C. (2008). Interactive Whiteboards: Real Beauty or Just “Lipstick”?. Comput. Educ..

[44] Šumak B., Pušnik M., Heričko M., Šorgo A. (2017). Differences between Prospective, Existing, and Former Users of Interactive Whiteboards on External Factors Affecting Their Adoption, Usage and Abandonment. Comput. Hum. Behav..

[45] Coyle Y., Yañez L., Verdú M. (2010). The Impact of the Interactive Whiteboard on the Teacher and Children’s Language Use in an ESL Immersion Classroom. System.

[46] Aldunate R., Nussbaum M. (2013). Teacher Adoption of Technology. Comput. Hum. Behav..

[47] Winzenried A., Dalgarno B., Tinkler J. (2010). The Interactive Whiteboard: A Transitional Technology Supporting Diverse Teaching Practices. Australas. J. Educ. Technol..

[48] Perugini M., Bagozzi R. (2001). The Role of Desires and Anticipated Emotions in Goal-Directed Behaviors: Broadening and Deepening the Theory of Planned Behavior. Br. J. Soc. Psychol..

[49] Ajzen I. (1991). The Theory of Planned Behavior. Organ. Behav. Hum. Decis. Process..

[50] Han H., Kim W., Lee S. (2018). Stimulating Visitors’ Goal-Directed Behavior for Environmentally Responsible Museums: Testing the Role of Moderator Variables. J. Destin. Mark. Manag..

[51] Ko H.-C. (2018). Social Desire or Commercial Desire? The Factors Driving Social Sharing and Shopping Intentions on Social Commerce Platforms. Electron. Commer. Res. Appl..

[52] Kim M.J., Hall C.M. (2021). Do Perceived Risk and Intervention Affect Crowdfunder Behavior for the Sustainable Development Goals? A Model of Goal-Directed Behavior. J. Clean. Prod..

[53] Su L., Yang X., Huang Y. (2022). How Do Tourism Goal Disclosure Motivations Drive Chinese Tourists’ Goal-Directed Behaviors? The Influences of Feedback Valence, Affective Rumination, and Emotional Engagement. Tour. Manag..

[54] Osakwe C.N., Hudik M., Říha D., Stros M., Ramayah T. (2022). Critical Factors Characterizing Consumers’ Intentions to Use Drones for Last-Mile Delivery: Does Delivery Risk Matter?. J. Retail. Consum. Serv..

[55] Dochow S., Neumeyer S. (2021). An Investigation of the Causal Effect of Educational Expectations on School Performance. Behavioral Consequences, Time-Stable Confounding, or Reciprocal Causality?. Res. Soc. Stratif. Mobil..

[56] Lee M.-C. (2010). Explaining and Predicting Users’ Continuance Intention toward e-Learning: An Extension of the Expectation–Confirmation Model. Comput. Educ..

[57] Zhou M. (2016). Chinese University Students’ Acceptance of MOOCs: A Self-Determination Perspective. Comput. Educ..

[58] Nie J., Zheng C., Zeng P., Zhou B., Lei L., Wang P. (2020). Using the Theory of Planned Behavior and the Role of Social Image to Understand Mobile English Learning Check-in Behavior. Comput. Educ..

[59] Watson J.H., Rockinson-Szapkiw A. (2021). Predicting Preservice Teachers’ Intention to Use Technology-Enabled Learning. Comput. Educ..

[60] Valtonen T., Kukkonen J., Kontkanen S., Sormunen K., Dillon P., Sointu E. (2015). The Impact of Authentic Learning Experiences with ICT on Pre-Service Teachers’ Intentions to Use ICT for Teaching and Learning. Comput. Educ..

[61] Sirisookslip S., Ariratana W., Ngang T.K. (2015). The Impact of Leadership Styles of School Administrators on Affecting Teacher Effectiveness. Procedia-Soc. Behav. Sci..

[62] Perugini M. (2004). An Alternative View of Pre-Volitional Processes in Decision Making: Conceptual Issues and Empirical Evidence. Contemporary Perspectives on the Psychology of Attitudes.

[63] Bagozzi R.P., Wong N., Yi Y. (1999). The Role of Culture and Gender in the Relationship between Positive and Negative Affect. Cogn. Emot..

[64] Miller Z.D. (2017). The Enduring Use of the Theory of Planned Behavior. Hum. Dimens. Wildl..

[65] Jiang F., Lu S., Hou Y., Yue X. (2013). Dialectical Thinking and Health Behaviors: The Effects of Theory of Planned Behavior. Int. J. Psychol..

[66] Topa G., Moriano J.A. (2010). Theory of Planned Behavior and Smoking: Meta-Analysis and SEM Model. Subst. Abuse Rehabil..

[67] El Khoury D., Tabakos M., Dwyer J.J.M., Mountjoy M. (2021). Determinants of Supplementation among Canadian University Students: A Theory of Planned Behavior Perspective. J. Am. Coll. Health.

[68] Goh E., Ritchie B. (2011). Using the Theory of Planned Behavior to Understand Student Attitudes and Constraints Toward Attending Field Trips. J. Teach. Travel Tour..

[69] Harding T.S., Mayhew M.J., Finelli C.J., Carpenter D.D. (2007). The Theory of Planned Behavior as a Model of Academic Dishonesty in Engineering and Humanities Undergraduates. Ethics Behav..

[70] Han H., Meng B., Kim W. (2017). Emerging Bicycle Tourism and the Theory of Planned Behavior. J. Sustain. Tour..

[71] Ramamonjiarivelo Z., Martin D.S., Martin W.S. (2015). The Determinants of Medical Tourism Intentions: Applying the Theory of Planned Behavior. Health Mark. Q..

[72] Alavion S.J., Allahyari M.S., Al-Rimawi A.S., Surujlal J. (2017). Adoption of Agricultural E-Marketing: Application of the Theory of Planned Behavior. J. Int. Food Agribus. Mark..

[73] Chu S.-C., Chen H.-T., Sung Y. (2016). Following Brands on Twitter: An Extension of Theory of Planned Behavior. Int. J. Advert..

[74] Steinmetz H., Knappstein M., Ajzen I., Schmidt P., Kabst R. (2016). How Effective Are Behavior Change Interventions Based on the Theory of Planned Behavior?. Z. Psychol..

[75] Ajzen I. (2011). The Theory of Planned Behaviour: Reactions and Reflections. Psychol. Health.

[76] Yim B.H., Byon K.K. (2021). Validation of the Sport Fan Model of Goal-Directed Behavior: Comparison to Theory of Reasoned Action, Theory of Planned Behavior, and Model of Goal-Directed Behavior. J. Glob. Sport Manag..

[77] Kwasnicka D., Dombrowski S.U., White M., Sniehotta F. (2016). Theoretical Explanations for Maintenance of Behaviour Change: A Systematic Review of Behaviour Theories. Health Psychol. Rev..

[78] Song H.J., Lee C.-K., Kang S.K., Boo S. (2012). The Effect of Environmentally Friendly Perceptions on Festival Visitors’ Decision-Making Process Using an Extended Model of Goal-Directed Behavior. Tour. Manag..

[79] Ajzen I., Driver B.L. (1992). Application of the Theory of Planned Behavior to Leisure Choice. J. Leis. Res..

[80] Perugini M., Bagozzi R.P. (2004). The Distinction between Desires and Intentions. Eur. J. Soc. Psychol..

[81] Fishbein M., Ajzen I. (1975). Belief, Attitude, Intention, and Behavior: An Introduction to Theory and Research.

[82] Davis F.D. (1989). Perceived Usefulness, Perceived Ease of Use, and User Acceptance of Information Technology. MIS Q..

[83] Chu T.-H., Chen Y.-Y. (2016). With Good We Become Good: Understanding e-Learning Adoption by Theory of Planned Behavior and Group Influences. Comput. Educ..

[84] Wu J.Y., Chen L.T. (2020). Odoo ERP with Business Intelligence Tool for a Small-Medium Enterprise: A Scenario Case Study. Proceedings of the 2020 11th International Conference on E-Education, E-Business, E-Management, and E-Learning.

[85] Shahidi N., Tossan V., Bourliataux-Lajoinie S., Cacho-Elizondo S. (2022). Behavioural Intention to Use a Contact Tracing Application: The Case of StopCovid in France. J. Retail. Consum. Serv..

[86] Bagozzi R.P., Dholakia U.M., Basuroy S. (2003). How Effortful Decisions Get Enacted: The Motivating Role of Decision Processes, Desires, and Anticipated Emotions. J. Behav. Decis. Mak..

[87] Leone L., Perugini M., Ercolani A.P. (2004). Studying, Practicing, and Mastering: A Test of the Model of Goal-Directed Behavior (MGB) in the Software Learning Domain. J. Appl. Soc. Psychol..

[88] Amaro S., Duarte P. (2016). Travellers’ Intention to Purchase Travel Online: Integrating Trust and Risk to the Theory of Planned Behaviour. Anatolia.

[89] Utami C.W. (2011). Analysis of Emotion, Habit, and Rational Choice: A Study on Consumer Behavior. Int. Res. J. Bus. Stud..

[90] Chen G., Zou M., Ran N., Yan B., Li S. (2023). The Effects of Environmental Empathy and Sustainable Intelligence on Wetland Tourists’ Revisit Intention Using an Extended Model of Goal-Directed Behavior. J. Clean. Prod..

[91] Kim M.-J., Lee M.J., Lee C.-K., Song H.-J. (2012). Does Gender Affect Korean Tourists’ Overseas Travel? Applying the Model of Goal-Directed Behavior. Asia Pac. J. Tour. Res..

[92] Malle B.F., Moses L.J., Baldwin D.A. (2001). Intentions and Intentionality: Foundations of Social Cognition.

[93] Fry M.-L., Drennan J., Previte J., White A., Tjondronegoro D. (2014). The Role of Desire in Understanding Intentions to Drink Responsibly: An Application of the Model of Goal-Directed Behaviour. J. Mark. Manag..

[94] Han H., Yoon H.J. (2015). Hotel Customers’ Environmentally Responsible Behavioral Intention: Impact of Key Constructs on Decision in Green Consumerism. Int. J. Hosp. Manag..

[95] Jung S.E., Santella M., Hermann J., Lawrence J. (2018). Understanding College Students’ Intention to Consume Fruits and Vegetables: An Application of the Model of Goal Directed Behavior. Int. J. Health Promot. Educ..

[96] Kim M.J., Preis M.W. (2016). Why Seniors Use Mobile Devices: Applying an Extended Model of Goal-Directed Behavior. J. Travel Tour. Mark..

[97] Piçarra N., Giger J.-C. (2018). Predicting Intention to Work with Social Robots at Anticipation Stage: Assessing the Role of Behavioral Desire and Anticipated Emotions. Comput. Hum. Behav..

[98] Ajzen I., Fishbein M. (1977). Attitude-Behavior Relations: A Theoretical Analysis and Review of Empirical Research. Psychol. Bull..

[99] Bagozzi R.P., Baumgartner H., Pieters R. (1998). Goal-Directed Emotions. Cogn. Emot..

[100] Lai Y., Saab N., Admiraal W. (2022). University Students’ Use of Mobile Technology in Self-Directed Language Learning: Using the Integrative Model of Behavior Prediction. Comput. Educ..

[101] Park E., Lee S., Peters D.J. (2017). Iowa Wetlands Outdoor Recreation Visitors’ Decision-Making Process: An Extended Model of Goal-Directed Behavior. J. Outdoor Recreat. Tour..

[102] Kaiser H.F., Rice J. (1974). Little Jiffy, Mark Iv. Educ. Psychol. Meas..

[103] Nunnally J.C., Bernstein I.H. (1994). Psychometric Theory.

[104] Son H., Park Y., Kim C., Chou J.-S. (2012). Toward an Understanding of Construction Professionals’ Acceptance of Mobile Computing Devices in South Korea: An Extension of the Technology Acceptance Model. Autom. Constr..

[105] Hair J.F., Black W.C., Babin B.J., Anderson R.E. (2013). Multivariate Data Analysis.

[106] Jöreskog K.G., Sörbom D. (2018). LISREL 10 for Windows [Computer Software].

[107] Lee C.-K., Song H.-J., Bendle L.J., Kim M.-J., Han H. (2012). The Impact of Non-Pharmaceutical Interventions for 2009 H1N1 Influenza on Travel Intentions: A Model of Goal-Directed Behavior. Tour. Manag..

